# The dose of hydroxyethyl starch 6% 130/0.4 for fluid therapy and the incidence of acute kidney injury after cardiac surgery: A retrospective matched study

**DOI:** 10.1371/journal.pone.0186403

**Published:** 2017-10-18

**Authors:** Mona Momeni, Lompoli Nkoy Ena, Michel Van Dyck, Amine Matta, David Kahn, Dominique Thiry, André Grégoire, Christine Watremez

**Affiliations:** 1 Department of Anesthesiology, Université Catholique de Louvain, Cliniques Universitaires Saint Luc, Brussels, Belgium; 2 Department of Perfusion Services, Université Catholique de Louvain, Cliniques universitaires Saint Luc, Brussels, Belgium; National Cancer Institute, UNITED STATES

## Abstract

The safety of hydroxyethyl starches (HES) is still under debate. No studies have compared different dosing regimens of HES in cardiac surgery. We analyzed whether the incidence of Acute Kidney Injury (AKI) differed taking into account a weight-adjusted cumulative dose of HES 6% 130/0.4 for perioperative fluid therapy. This retrospective cohort study included all adult patients undergoing elective or emergency cardiac surgery with or without cardiopulmonary bypass. Exclusion criteria were patients on renal replacement therapy (RRT), cardiac trauma surgery, heart transplantation, patients with ventricular assist devices, subjects who required a surgical revision for bleeding and those whose medical records were incomplete. Primary endpoint was AKI following the creatinine based RIFLE classification. Secondary endpoints were 30-day mortality and RRT. Patients were divided into 2 groups whether they had received a cumulative HES dose of < 30 mL/kg (Low HES) or ≥ 30 mL/kg (High HES) during the intra- and postoperative period. A total of 1501 patients were analyzed with 983 patients in the Low HES and 518 subjects in the High HES group. 185 (18.8%) patients in the Low HES and 119 (23.0%) patients in the High HES group developed AKI (P = 0.06). In multivariable regression analysis the dose of HES administered per weight was not associated with AKI. After case-control matching 217 patients were analyzed in each group. AKI occurred in 39 (18.0%) patients in the Low HES and 50 (23.0%) patients in the High HES group (P = 0.19). In conditional regression analysis performed on the matched groups a lower weight-adjusted dose of HES was significantly associated with a reduced incidence of AKI [(Odds Ratio (95% CI) = 0.825 (0.727–0.936); P = 0.003]. In the absence of any safety study the cumulative dose of modern HES in cardiac surgery should be kept less than 30 mL/kg.

## Introduction

Fluid resuscitation with hydroxyethyl starch (HES) has been associated with an increased risk of renal—replacement therapy (RRT) [1, 2] and/or mortality in critically ill patients admitted to the intensive care unit (ICU) [3]. These trials showing harmful effects of HES have mainly evaluated a non-surgical population [1–3]. The VISEP study [1] and the 6S trial [2] compared HES and crystalloids in ICU patients with severe sepsis. In the CHEST trial HES was compared with crystalloids in a heterogeneous group of patients treated in the ICU [3]. In their trial 42,5% of patients in the HES group and 42,9% of patients in the saline group were surgical cases. However, the pathophysiology of renal failure in a non-surgical population differs from patients undergoing surgery [4, 5].

Patients undergoing cardiac surgery belong furthermore to a particular surgical population at high risk of developing acute kidney injury (AKI) [6]. Cardiac surgery-associated acute kidney injury can occur in up to 30% of the patients [7–9], and is associated with an increased incidence of mortality [10–12]. The results of a recent retrospective study suggested that using HES 6% 130/0.4 for cardiopulmonary bypass (CPB) prime and intraoperative fluid therapy at an average dose of 30 mL/kg was associated with a greater incidence of AKI after on-pump adult cardiac surgery when compared with fluid therapy based on crystalloids only [13]. To date no study has evaluated the association between the total dose of HES 6% 130/0.4 administered in the perioperative period and the incidence of AKI after adult cardiac surgery. The aim of this retrospective analysis was to seek whether the incidence of in-hospital AKI was different when taking into account a weight-adjusted cumulative dose of HES administered for perioperative fluid therapy.

## Materials and methods

This was a retrospective cohort study. The review of the patients’ medical records was approved on October the 27^th^ 2014 by «La Commission d’Ethique Hospitalo-Facultaire de l’UCL» in Brussels, Belgium (2014, 505).

We analyzed the data of all patients > 18 years who underwent elective or emergency cardiac surgery with or without CPB between January 2011 and April 2013. The collection of the data was realized between July 2014 and September 2014. Exclusion criteria were as follows: patients with preoperative RRT, any trauma patients requiring emergency cardiac surgery, heart transplantation patients, subjects who were put on extracorporeal life support or long-term ventricular assist devices, all patients who necessitated a surgical revision for bleeding and/or tamponade after initial cardiac surgery and patients whose medical records were incomplete.

The intraoperative and postoperative management of the patients were standardized according to the institutional's guidelines. Anesthesia was induced with midazolam, sufentanil, ketamine and propofol and continued with either propofol or sevoflurane. Muscle relaxation was achieved with rocuronium. All patients received cefazolin as antibiotic prophylaxis. In addition to standard hemodynamic monitoring, a transesophageal echocardiography and/or a pulmonary artery catheter were used in selected cases. Packed red blood cells (RBC) were transfused to achieve a hematocrit of ≥ 20% on CPB and a hematocrit of ≥ 25% after separation from CPB unless signs of poor tolerance developed. The transfusion of non-red blood cell components was performed only if clinical bleeding was present in conjunction with abnormal point-of-care coagulation tests. Clinical bleeding was also diagnosed if the surgeons could not visualize clots in the surgical field after protamine administration. Tranexamic acid was used in all patients with a maximum dose of 30 mg/kg. Half of this dose was administered at the induction of anesthesia and half was given in the CPB machine. This dose was decreased in case of chronic kidney disease. Patients were transferred to a cardiac ICU and were kept sedated with propofol until hemodynamically stable and ready to be extubated. The same senior surgeons, cardiac anesthesiologists and cardiac intensivists managed the patients during the entire study period.

### Cardiopulmonary bypass management

Routine normothermic CPB at a standardised continuous nonpulsatile flow of 2.4/ L/min/m^2^ was performed. Myocardial protection was achieved with warm blood enriched with potassium chloride and magnesium sulfate. In patients undergoing aortic arch surgery or aortic dissection, moderate hypothermia (28°C) with circulatory arrest and anterograde selective cerebral perfusion was performed. CPB prime varied slightly and consisted of 1000 mL Ringer Acetate Solution (Plasmalyte A^®^, Baxter, S.A. Lessines, Belgium) with a mixture of either 500 mL Ringer Acetate Solution or 500 mL HES 6% 130/0.4 (Voluven^®^ or Volulyte^®^, Fresenius Kabi, Belgium), together with 100 mg of heparin and 2 mL/kg of Mannitol 15%. No steroids were injected. CPB management was the responsibility of the cardiac anesthesiologist. As such, additional fluids used during the CPB slightly varied depending on the anesthesiologist in charge of the patient who adapted the fluid therapy in function of the patient's pathology.

### Outcome measures

Our primary endpoint was AKI. The secondary endpoints were 30-day mortality and RRT. We defined AKI according to the serum creatinine (sCreat) criterion of the renal RIFLE (Risk, Injury, Failure, Loss of renal function and End-stage renal disease) classification [14]. We considered peak values of sCreat within the first seven postoperative days or the entire hospital stay if shorter. The criteria that led to the worst possible classification were used. In patients with chronic kidney disease in who the increase in sCreat was under threefold, the F component of the RIFLE criteria was used as long as there was an acute increase of at least 0.5 mg/dL and the new sCreat was greater than 4.0 mg/dL. For patients in the R component of RIFLE criteria, in addition to the percentage change from baseline sCreat, we also added the criterion of an absolute sCreat increment of ≥ 0.3 mg/dL. This was performed in order to include minimal changes of sCreat. It has been previously demonstrated that even minimal changes of sCreat can predict poor prognosis in patients after cardiac surgery [10]. Because of the retrospective nature of this study, data on the urine output were not completely available and as such the urine ouput criteria of the RIFLE classification were not applied. To be noted, the creatinine only-based criterion is not influenced by diuretic use and has been shown to be superior at predicting mortality [15].

### Fluid administration

The intra- and postoperative volume therapy were left at the discretion of the physician in charge of the patient. The type and the cumulative dose of all administered crystalloids, colloids and blood products were recorded for the intraoperative period (fluids administered during anesthesia—fluids given during CPB including the priming solution) and for the entire postoperative stay in the ICU. Patients were arbitrarily divided into 2 groups according to the cumulative dose of HES 6% 130/0.4 they had received during the entire intra- and postoperative period. Patients in the «Low HES» group had received a cumulative HES dose of < 30 mL/kg and patients in the «High HES» group had received a cumulative HES dose of ≥ 30 mL/kg. We performed a supplementary analysis to clarify whether the risk of AKI was increased in a dose-dependent manner by making a comparison between patients who had received a cumulative HES dose of < 30 mL/kg, those who had received a cumulative HES dose between 30–50 mL/kg and those who had received a cumulative HES dose of ≥ 50 mL/kg. The latter value is the dose limit proposed by the manufacture.

### Statistical analysis

The distribution of the data was tested using Smirnov-Kolmogorov test. All data are expressed as median (Percentile 25—Percentile 75), minimum—maximum and numbers and percentages as appropriate. A Mann-Whitney and Chi-square test were used to respectively compare continuous and categorical variables. Fisher's exact test was used when applicable. Baseline and clinical variables showing a statistically significant difference between both groups or almost statistically significant difference were entered into a logistic regression analysis. Univariable and multivariable binary logistic regression analysis using enter method were applied to evaluate the association between the predefined AKI (regardless of the stage of RIFLE classification) and the cumulative dose of HES, the group assignment and the aforementioned covariates. The Hosmer-Lemeshow goodness-of-fit test and the Likelihood-ratio test were used to test the validity of the logistic regression model. To account for differences in baseline and clinical characteristics, a case-control matching was realized. The fuzzy algorithm for matching without replacement was used. Balance of the continuous covariables was evaluated using the standardized mean difference (SMD). A SMD > 0.1 was considered indicative of poor matching. A McNemar test and a Wilcoxon paired test were used to respectively compare categorical and quantitative variables between the matched groups. A conditional logistic regression analysis was carried out on the entire matched population to evaluate the association between the incidence of AKI (regardless of the stage of RIFLE classification) and the cumulative volume of HES administered, together with the covariates for which the matched groups showed a statistically significant difference.

Considering that the incidence of AKI after cardiac surgery is around 20% and this was considered to be the case in the Low HES group, and taking into account an increase of 5% as clinically significant, a total number of 210 patients were required in each group with a power of 80% and a one-sided α error set at 0.05.

All p values were two-sided and were considered to be statistically significant if < 0.05. Statistical analysis was carried out using IBM^®^ SPSS^®^ Statistics Software Package version 23.

## Results

**Fig 1:**
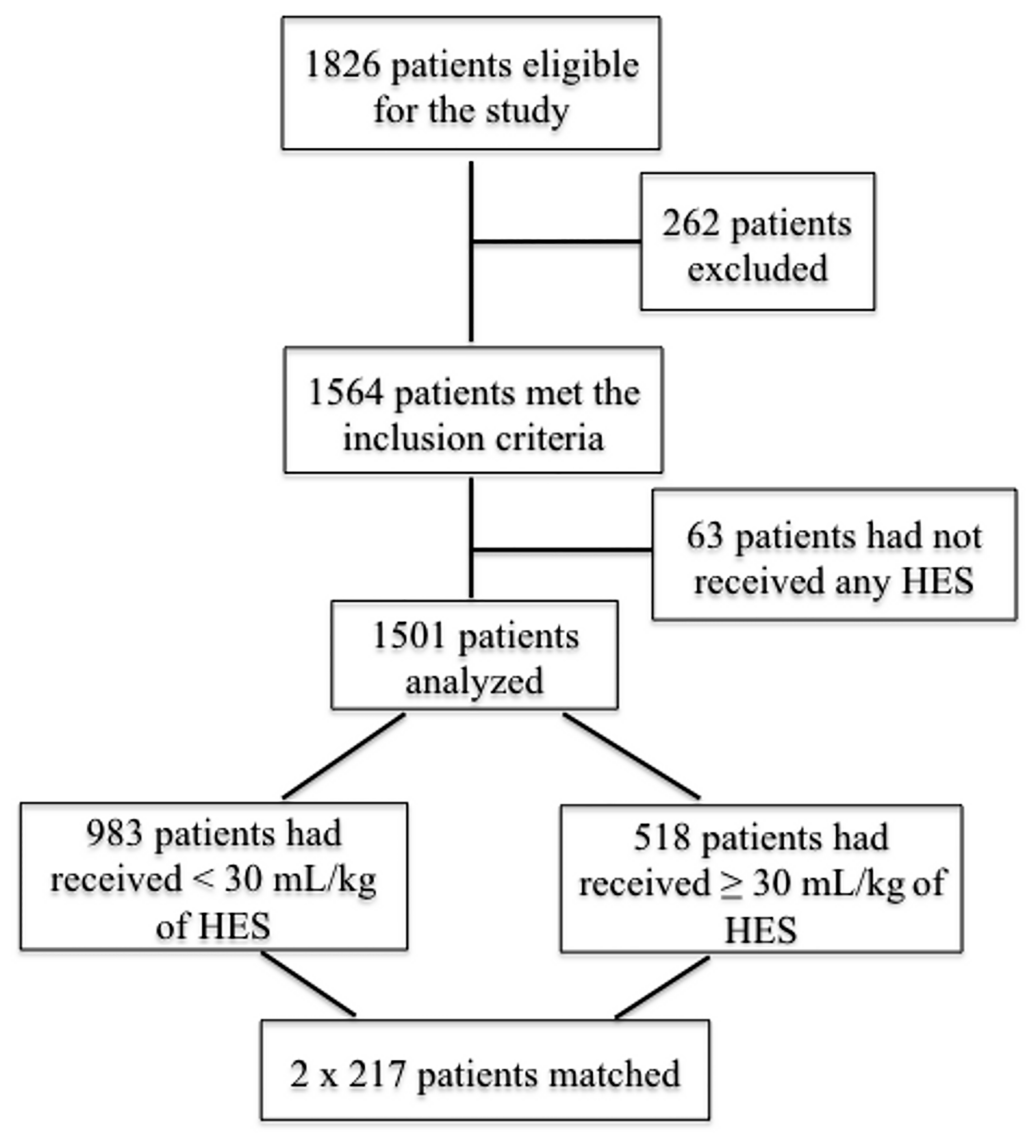
Flow chart of the study population. Patients who received a cumulative dose of < 30 mL/kg of HES (hydroxyethyl starch) 6% 130/ 0.4 were compared with those who had received a cumulative dose of ≥ 30 mL/kg of HES 6% 130/ 0.4.

During the study period 1826 patients underwent a cardiac surgery. In total the data of 1501 patients were reviewed as 63 patients had not received any HES solution and 262 patients did not meet the inclusion criteria. 983 subjects had received a low dose of HES and 518 patients were in the High HES group. Fig 1 shows the flowchart of the study.

The baseline characteristics of the studied population are shown in Table 1.

**Table 1:** Preoperative characteristics of the entire group and the matched population.

	Low HES (N = 983)	High HES (N = 518)	P value	Low HES (N = 217) Matched	High HES (N = 217) Matched	P value
**Age (years)**	68 (58–77)	71 (61–79)	0.006	68 (59–77)	69 (60–78)	0.27
**EuroSCORE II (%)**	2.09 (1.13–4.49)	2.44 (1.31–5.00)	0.03	2.03 (0.96–3.99)	2.14 (1.22–4.82)	0.35
**Creatinine (mg/dL)**	0.96 (0.80–1.15)	0.94 (0.80–1.10)	0.07	0.91 (0.79–1.11)	0.94 (0.81–1.10)	0.49
**Hemoglobin (g/dL)**	12.2 (10.9–13.5)	12.0 (10.5–13.10)	0.006	12.2 (11.1–13.3)	12.0 (10.9–13.2)	0.72
**Male sex**	677 (68.9%)	312 (60.2%)	0.001	136 (62.7%)	136 (62.7%)	1.0
**Ca**^**2+**^ **antagonists**	180 (18.3%)	107 (20.7%)	0.27	51 (23.5)	44 (20.3)	0.48
**ACE inhibitors**	532 (54.1%)	259 (50.0%)	0.13	114 (52.5%)	105 (48.4%)	0.43
**Diuretics**	369 (37.5%)	164 (31.7%)	0.02	73 (33.6%)	73 (33.6%)	1.0
**Beta blockers**	543 (55.2%)	299 (57.7%)	0.24	118 (54.4%)	120 (55.3%)	0.59
**Aspirin**	583 (59.3%)	331 (63.9%)	0.08	124 (57.1%)	138 (63.6%)	0.18
**Clopidogrel**	90 (9.2%)	44 (8.5%)	0.70	15 (6.9%)	19 (8.8%)	0.59

HES: hydroxyethyl starch. EuroSCORE: European System for Cardiac Operative Risk Evaluation. ACE: angiotensin converting enzyme. Data are expressed as median (Percentile 25 -Percentile 75) or numbers (percentages).

The intraoperative data of the groups are illustrated in Table 2.

**Table 2:** Intra- and postoperative data of the entire group and the matched population.

	Low HES (N = 983)	High HES (N = 518)	P value	Low HES (N = 217) Matched	High HES (N = 217) Matched	P value
**Elective surgery**	926 (94.2%)	502 (96.9%)	0.02	214 (98.6%)	214 (98.6%)	1.0
**On-pump surgery**	826 (84.0%)	456 (88.0%)	0.04	193 (88.9%)	193 (88.9%)	1.0
**IABP**	15 (1.5%)	4 (0.8%)	0.21	2 (0.9%)	1 (0.5%)	0.99
**CPB time (minutes)**	92 (60–127)	98 (67–130)	0.06	91 (63–117)	101 (73–131)	0.02
**ACC time (minutes)**	64 (41–98)	69 (47–101)	0.08	63 (45–91)	72 (51–106)	0.05
**Reinfused cell-saver (mL)**	605 (480–750)	611 (496–756)	0.40	631 (474–820)	581 (485–736)	0.50
**Cardiac procedure**			0.33			0.45
**CABG only**	283 (28.7%)	154 (29.7%)		58 (26.7%)	67 (30.9%)	
**Valve only**	359 (36.5%)	168 (32.4%)		85 (39.2%)	71 (32.7%)	
**CABG + valve**	116 (11.8%)	73 (14.1%)		24 (11.1%)	28 (12.9%)	
**CABG + other**	20 (2.0%)	17 (3.3%)		2 (0.9%)	6 (2.8%)	
**Other**	31 (3.1%)	13 (2.5%)		6 (2.8%)	7 (3.2%)	
**Valve + other**	151 (15.3%)	75 (14.5%)		39 (18.0%)	31 (14.3%)	
**CABG + valve + other**	23 (2.3%)	18 (3.5%)		3 (1.4%)	7 (3.2%)	
**ICU stay (days)**	2.15 (1.87–3.14)	2.29 (1.97–4.11)	< 0.001	2.14 (1.87–3.05)	2.29 (1.92–4.10)	0.009
**Hospital stay (days)**	9 (7–13)	9 (7–15)	< 0.001	8 (7–13)	9 (8–14)	0.01
**Mortality**	30 (3.1%)	24 (4.6%)	0.12	5 (2.3%)	6 (2.8%)	0.99
**Peak creatinine (mg/dL)**	1.00 (0.82–1.31)	1.03 (0.82–1.34)	0.72	0.93 (0.77–1.22)	1.04 (0.82–1.33)	0.002
**RIFLE criteria present**			0.06			0.19
**Any**	185 (18.8%)	119 (23.0%)		39 (18.0%)	50 (23.0%)	
**R**	113 (11.5%)	78 (15.1%)		25 (11.5%)	31 (14.3%)	
**I**	28 (2.8%)	12 (2.3%)		6 (2.8%)	8 (3.7%)	
**F**	17 (1.7%)	7 (1.4%)		3 (1.4%)	2 (0.9%)	
**L**	27 (2.7%)	21 (4.1%)		5 (2.3%)	9 (4.1%)	
**E**	0	1 (0.2%)		0	0	
**RIFLE criteria L & E**	27 (2.7%)	22 (4.2%)	0.12	5 (2.3%)	9 (4.1%)	0.34

HES: hydroxyethyl starch. IABP: intra-aortic balloon pump. CPB: cardiopulmonary bypass. ACC: aortic cross-clamp. CABG: coronary artery bypass graft. ICU: intensive care unit. RIFLE (Risk, Injury, Failure, Loss of renal function and End-stage renal disease) criteria. Data are expressed as median (Percentile 25 -Percentile 75) or numbers (percentages).

Elective surgery and on-pump surgery were performed in a significantly higher number of patients in the High HES group as compared with the Low HES group (respectively 0.02 and 0.04). The postoperative data are shown in Table 2. AKI occurred in more patients in the High HES group (23%) compared to the low HES group (18.8%) although this difference did not reach statistical significance (P = 0.06). RRT was used in 4.2% of the patients in the High HES group and 2.7% of the patients in the Low HES group (P = 0.12). There was no significant difference in the 30-day mortality between both groups (P = 0.12).

The supplementary analysis revealed in addition to the 518 patients who had received a cumulative HES dose of < 30 mL/kg, 442 patients who had received a cumulative HES dose of 30–50 mL/kg of whom 94 (21.3%) suffered from AKI and 76 patients who had received a cumulative HES dose ≥ 50 mL/kg of whom 25 (32.9%) showed AKI. The incidence of AKI was significantly different between these 3 groups (P = 0.011; significant after Post Hoc Bonferroni test). A receiver operating curve was analyzed with the aim to calculate a possible cutoff value at which cumulative amount of HES AKI would occur. However, the area under the curve was only 0.545 (P = 0.02).

Data on the intraoperative and postoperative fluid therapy are illustrated in Table 3.

**Table 3:** Fluid therapy of the entire group and the matched population.

	Low HES (N = 983)	High HES (N = 518)	P value	Low HES (N = 217) Matched	Hgh HES (N = 217) Matched	P value
**N patients who received HES**						
Intraoperatively*	670 (68.2%)	504 (97.3%)	< 0.001	144 (66.4%)	211 (97.2%)	< 0.001
**Postoperatively**	907 (92.3%)	514 (99.2%)	< 0.001	210 (96.8%)	216 (99.5%)	0.03
**Total volume HES administered**	1500 (1000–2000)	2500 (2500–3000)	< 0.001	1500 (1000–2000)	2500 (2500–3000)	< 0.001
**Intraoperatively**	500 (0–1000)	1500 (1000–1500)	< 0.001	500 (0–1000)	1500 (1000–1500)	< 0.001
**Postoperatively**	1000 (500–1000)	1500 (1000–2000)	< 0.001	1000 (500–1000)	1500 (1000–1500)	< 0.001
**Volume HES per weight (mL/kg)**	18 (13–24)	38 (34–45)	< 0.001	18 (13–25)	38 (34–44)	< 0.001
**N patients who received** **HES via CPB machine**	510 (61.7% = 510/826)	434 (95.2% = 434/456)	< 0.001	117 (60.6% = 117/193)	186 (96.4% = 186/193)	< 0.001
**Volume HES administered via CPB machine**	500 (0–500)	500 (500–1000)	< 0.001	500 (0–500)	900 (500–1000)	< 0.001
**N patients who received** **HES during anesthesia**	320 (32.6%)	373 (72.0%)	< 0.001	57 (26.3%)	153 (70.5%)	< 0.001
**Volume HES administered during anesthesia**	0 (0–500)	500 (0–1000)	< 0.001	0 (0–500)	500 (0–1000)	< 0.001
**N patients who received gelatins intra- & postoperatively**	874 (88.9%)	509 (98.3%)	< 0.001	202 (93.1%)	211 (97.2%)	0.08
**N patients who received gelatins via CPB machine**	132 (16.0% = 132/826)	80 (17.5% = 80/456)	0.48	24 (12.4% = 24/193)	42 (21.8% = 42/193)	0.03
**Volume Gelatins administered via CPB machine** Min—Max**	0 (0–0) 0–2500**	0 (0–0) 0–1500**	0.50	0 (0–0) 0–1400**	0 (0–0) 0–1000**	0.05
**Volume Gelatins administered postoperatively**	1000 (500–1000)	1000 (1000–1500)	< 0.001	1000 (500–1000)	1000 (1000–1500)	< 0.001
**Volume crystalloids administered during anesthesia**	1300 (1000–1700)	1200 (1000–1700)	0.42	1500 (1000–2000)	1200 (1000–1700)	0.20
**Volume crystalloids administered via CPB machine**	2300 (2000–3500)	2000 (2000–2500)	< 0.001	2300 (2000–3500)	2000 (2000–2300)	< 0.001
**Volume crystalloids administered postoperatively** Min—Max**	0 (0–0) 0–4000**	0 (0–0) 0–1500**	< 0.001	0 (0–0) 0–2000**	0 (0–0) 0–1000**	0.37
**N patients receiving albumin postoperatively**	14 (1.4%)	29 (5.6%)	< 0.001	2 (0.9%)	10 (4.6%)	0.04

HES: hydroxyethyl starch. CPB: cardiopulmonary bypass. Volumes are represented in mL.

*: during anesthesia as well as via cardiopumonary bypass machine. Data are expressed as median (Percentile 25 -Percentile 75) or numbers (percentages) and

** Min—Max.

The minimum volume of HES administered in the Low HES group was 2.87 mL/kg. The maximum volume of HES administerd in the High HES group was 90.16 mL/kg. Significantly more patients in the High HES group required significantly more allogeneic blood products of any kind as illustrated in Table 4.

**Table 4:** Transfusion data of the entire group and the matched population.

	Low HES (N = 983)	High HES (N = 518)	p value	Low HES (N = 217) Matched	High HES (N = 217) Matched	P value
**Patients who received RBC intraoperatively**	307 (31.2%)	249 (48.1%)	< 0.001	67 (30.9%)	98 (45.2%)	0.001
**Units RBC transfused intraoperatively**	0–18	0–25	< 0.001	0–15	0–25	0.002
**Patients who received FFP intraoperatively**	124 (12.6%)	84 (16.2%)	0.06	23 (10.6%)	38 (17.5%)	0.05
**Units FFP transfused intraoperatively**	0–21	0–22	0.07	0–17	0–15	0.43
**Patients who received platelets intraoperatively**	140 (14.2%)	98 (18.9%)	0.02	24 (11.1%)	42 (19.4%)	0.03
**Units platelets transfused intraoperatively**	0–78	0–70	0.01	0–60	0–70	< 0.001
**Patients who received RBC postoperatively**	128 (13.0%)	194 (37.5%)	< 0.001	25 (11.5%)	86 (39.6%)	< 0.001
**Units RBC transfused postoperatively**	0–11	0–9	< 0.001	0–4	0–8	< 0.001
**Patients who received FFP postoperatively**	47 (4.8%)	74 (14.3%)	< 0.001	12 (5.5%)	35 (16.1%)	< 0.001
**Units FFP transfused postoperatively**	0–9	0–8	< 0.001	0–3	0–5	0.001
**Patients who received platelets postoperatively**	62 (6.3%)	111 (21.4%)	< 0.001	12 (5.5%)	42 (19.4%)	< 0.001
**Units platelets transfused postoperatively**	0–30	0–30	< 0.001	0–15	0–30	< 0.001
**Total units RBC transfusedintra- and postoperatively**	0 (0–2)	1 (0–3)	< 0.001	0 (0–1)	1 (0–3)	< 0.001

HES: hydroxyethyl starch. RBC: red blood cells. FFP: fresh frozen plasma. Data are expressed as minimum—maximum, median (Percentile 25—Percentile 75) or numbers (percentages).

Univariable predictors of AKI for the whole group are presented in Table 5.

**Table 5:** Univariable and multivariable predictors of acute kidney injury for the entire group and the matched population.

	Univariable analysis Overall patients OR (95% CI)	P value	Multivariable analysis Overall patients OR (95% CI)	P value	Multivariable analysis Matched group OR (95% CI)	P value
**Grouping: Low vs high dose HES**	1.286 (0.992–1.668)	0.06	0.950 (0.562–1.606)	0.85		
**Dose HES per weight (mL/kg)**	1.013 (1.004–1.022)	0.005	1.016 (0.996–1.036)	0.11	0.825 (0.727–0.936)	0.003
**Age (years)**	1.023 (1.013–1.033)	< 0.001	1.009 (0.999–1.019)	0.07		
**EuroSCORE II (%)**	1.056 (1.038–1.074)	< 0.001	1.009 (0.981–1.038)	0.53		
**Preoperative creatinine (mg/dL)**	3.867 (2.748–5.441)	< 0.001	2.819 (1.774–4.480)	< 0.001		
**Preoperative hemoglobin (g/dL)**	0.757 (0.698–0.820)	< 0.001	0.834 (0.752–0.926)	0.001		
**Gender Male**	0.876 (0.674–1.139)	0.32	1.169 (0.814–1.678)	0.4		
**Preoperative diuretics**	0.535 (0.414–0.691)	< 0.001	0.873 (0.631–1.208)	0.41		
**Preoperative aspirin**	1.019 (0.788–1.319)	0.88	1.060 (0.771–1.459)	0.72		
**Elective surgery**	2.439 (1.490–3.993)	< 0.001	1.504 (0.770–2.935)	0.23		
**Total CPB time (minutes)**	1.006 (1.004–1.008)	< 0.001	1.009 (1.003–1.014)	0.004	1.011 (0.986–1.037)	0.38
**Total ACC time(minutes)**	1.003 (1.001–1.006)	0.01	0.998 (0.992–1.004)	0.46		
**Total units RBC transfused**	1.283 (1.216–1.355)	< 0.001	1.114 (1.024–1.211)	0.01	0.931(0.512–1.693)	0.82

OR: odds ratio. CI: confidence interval. HES: hydroxyethyl starch. CPB: cardiopulmonary bypass. ACC: aortic cross-clamp. RBC: red blood cells.

The cumulative dose of HES per weight was significantly associated with AKI in univariable analysis. However, this was not the case after adjustment for confounders in the multivariable analysis. In multivariable analysis baseline high sCreat, baseline low hemoglobin, the total duration of the CPB time and the total units red blood cells transfused in the intra- and postoperative period were the only predictors of AKI. The Hosmer-Lemeshow goodness-of-fit test was positive indicating that the model was well fitted (Chi-square = 3,740; P = 0.880).

### Case-control matched analysis

Case-control matched analysis resulted in 217 patients in each group. Matching was performed for the following characteristics: Age, baseline sCreat, gender, preoperative use of diuretics, elective surgery and on-pump surgery. Exact matching was performed for categorical variables. For continuous variable age a tolerance of 10 years and for continuous variable baseline sCreat a tolerance of 0.5 mg/dL were accepted. The SMD for age, baseline sCreat, EuroSCORE (European System for Cardiac Operative Risk Evaluation) II and baseline hemoglobin were respectively 0.03, 0.04, 0.09 and -0.03. Matching resulted in similar baseline characteristics (Table 1). However, after matching the CPB time was significantly longer in patients in the High HES group (Table 2). The postoperative data of the matched groups are represented in Table 2. The peak postoperative sCreat was significantly higher in the High HES group compared to the Low HES group (P = 0.002) although no significant difference was observed (P = 0.19) between the number of patients presenting AKI in the high HES group (23%) compared with the Low HES group (18%). The number of patients put on RRT was not significantly different (P = 0.34) between the High HES group (4.1%) and the Low HES group (2.3%). The 30-day mortality was 2.8% in the High HES group and 2.3% in the Low HES group (P = 0.99). Data on the fluid therapy and the transfusion of allogeneic blood products are illustrated in respectively Tables 3 and 4. Patients in the Low HES group had received a median HES dose of 18 mL/kg and patients in the High HES group had received a median HES dose of 38 mL/kg. Again, the transfusion of all allogeneic blood products was significantly higher in patients who had received a cumulative high dose of HES in the intra- and the postoperative period. Table 5 illustrates the results of the conditional multivariable regression analysis on the matched population. In addition to the covariate volume of HES administered per weight, the CPB time and the total units of red blood cells transfused were specified for inclusion in the model as these variables were significantly different between both matched groups. The model was well fitted: -2 Log Likelihood = 4,957 with a Chi-square = 30,691 and P = 0.000. A lower weight-adjusted dose of HES was significantly associated with a reduced odds of AKI [(Odds Ratio (95% CI) = 0.825 (0.727–0.936); P = 0.003] compared with a higher weight-adjusted dose. The total CPB time and the total units of transfused red blood cells were not associated with AKI.

## Discussion

This retrospective analysis of 1501 patients who underwent cardiac surgery evaluated whether the incidence of in-hospital AKI based on the creatinine criterion of RIFLE classification was different between patients who had received a cumulative HES dose of < 30 mL/kg compared with patients who had received a dose of ≥ 30 mL/kg during the intraoperative period and their postoperative stay in the ICU. After matching there was no significant difference in the incidence of AKI between both groups. However, in multivariable analysis conducted on the matched population, a lower administered volume of HES per kg of weight was significantly associated with a reduced incidence of AKI (Odds Ratio; 95% CI: 0.825; 0.727 to 0.936; P = 0.003). Furthermore, a subanalysis of the groups showed that patients who had received a cumulative HES dose of ≥ 50 mL/kg showed a higher incidence of AKI compared with patients receiving a cumulative HES dose of < 30 mL/kg and a HES dose of 30–50 mL/kg.

To our knowledge, no study in cardiac surgery has evaluated the incidence of AKI taking into account different doses of modern HES used for perioperative fluid therapy. Moreover, despite concerns regarding adverse renal function with the administration of HES, no randomized clinical trial in cardiac surgery has evaluated the association of HES 6% 130/0.4 and AKI based on RIFLE classification. The only randomized trial after cardiac surgery that has used RIFLE classification was performed with 10% solution of Pentastarch [16]. The authors did not observe an increased incidence of renal dysfunction with the use of a medium molecular size HES. However, the primary endpoint of the study was not AKI. Moreover, all their patients had received 750 mL of HES in the CPB prime. In a meta-analysis in patients undergoing cardiac surgery the authors looked at the impact of various HES generations on safety and efficacy endpoints [17]. Firm conclusions could not be drawn regarding AKI because of a lack of specific definition in most of these studies. Bayer et al. reported the clinical outcomes of 6478 patients undergoing cardiac surgery between 2004 and 2010 [18]. They divided the subjects into 3 groups whether HES 6% 130/0.4 or 4% gelatin or only crystalloids were given as fluid therapy in the operating room and in the ICU. They observed an increased risk of the stage Failure of RIFLE in patients who had received HES and gelatins. They also showed an increased use of RRT in the HES and gelatin groups compared with the crystalloid group. These results persisted after propensity score stratification. Although the authors changed the main fluid therapy over a six year period, the CPB prime over the entire period consisted of 500 to 1000 mL of HES. As such subjects in their crystalloid period had received a median cumulative dose of 8 mL/kg of a HES solution. In our study these patients belong to the "Low HES" group. On the other hand the median cumulative HES dose in their HES period was 36 mL/kg. These values correspond to our "High HES" group. In this regard their results are not conclusive. In a very recent retrospective study in cardiac surgery, the authors showed that the use of balanced HES 6% 130/0.4 as a CPB prime and for intraoperative fluid therapy was associated with a greater incidence of AKI based on the Acute Kidney Injury Network Classification [19] when compared with a balanced crystalloid solution [13]. Their conclusions are in line with a large Cochrane review that recommends alternate volume replacement therapy in place of HES [20]. In the study conducted by Lagny et al. [13] HES patients received a total dose of HES ranging from 2000 mL to 2500 mL (average dose around 30 mL/kg) during the short time period of the surgery. The administration of large doses of exogenous solutes such as HES over a short time period may result in osmotic nephrosis [21]. It is now known that different exogenous agents enter the tubular cells by means of pinocytosis. The pinocytic vacuoles fuse with each other and with proximal tubular lysosomes to form vacuoles that contain the exogenous agent and the hydrolytic enzymes. The exogenous agent is digested by lysosomal enzymes. However, in case large doses of an exogenous agent e.g. HES are given together with preexisting kidney dysfunction and/or hypoxia/ischemia, lysosomal digestion and degradation of vacuoles are impaired [21]. High doses of HES administered over a short time period in conjuction with a situation of ischemia/reperfusion, as is the case in surgrey with CPB, may explain the results obtained in the study conducted by Lagny et al. [13] In our study the total volume of HES was administered over a much longer time period as compared with the study conducted by Lagny et al. Nevertheless, in multivariable analysis we found that a larger dose of HES was associated with an increased incidence of AKI. This is an important finding as studies have not been conclusive regarding a safe dose of HES [20]. Indeed, fluids are drugs [22]. Their accurate dosing can mitigate their toxicity as is the case for any medication.

In our study there was no significant difference in mortality (P = 0.99) and in the number of patients put on RRT (P = 0.34) between matched patients in the Low HES group compared with the High HES group. These results are in line with statements that avoiding hypervolemia rather than just abandoning any kind of volume therapy might influence the patients' outcome [16, 23, 24]. In this study significantly more patients received significantly more allogeneic blood products in the high HES group. These results should be interpreted with caution as we excluded all patients who required surgical re-exploration. One explanation for this observation would be that these patients had received a high dose of HES as fluid resuscitation just because they presented with a hypovolemic condition due to more surgical bleeding. Therefore, any bias regarding this issue cannot be neglected. Moreover, the primary endpoint of this study was AKI and not postoperative bleeding.

### Limitations of the study

This study lacked a matched control group that had not received any HES. Only 63 patients had not received any HES solution during the study period. Therefore they were not entered in the analysis. Second, we did not use the urinary output criteria of the RIFLE classification. This may have an impact on the staging of the patients to a particular RIFLE classification. Nevertheless, we considered even minimal increases of sCreat levels (0.3 ≥ mg/dL) and as such the sensitivity to detect any AKI has been increased. A third point that can be considered as a bias is that we excluded all patients who necessitated a surgical revision for bleeding and/or tamponade. In our view these patients can show severe hemodynamic instability putting them at high risk of renal hypoperfusion. In addition, they often require transfusion of allogeneic blood products. As a matter of fact perioperative red blood cell transfusions and surgical reexploration have been shown to be strongly and independently associated with AKI after cardiac surgery [12]. The retrospective character of this study did not allow us to compare the hemodynamic parameters between both groups which is of course an important drawback of this study. Lastly, our results are based on the total body weight instead of the ideal body weight. However, this is rarely realized in most cases in adult surgery.

## Conclusions

This retrospective study in cardiac surgery compared the incidence of in-hospital AKI in function of the cumulative dose of HES 6% 130/ 0.4 administered for intra- and postoperative fluid resuscitation. After matching and in multivariable analysis, a cumulative volume of ≥ 30 mL/kg HES was significantly associated with an increased odds ratio of AKI. In the setting of the perioperative care of cardiac surgical patients fluid resuscitation with modern HES should be weight-adjusted and given at a maximum dose less than 30 mL/kg.
